# Discovering Distinct Functional Modules of Specific Cancer Types Using Protein-Protein Interaction Networks

**DOI:** 10.1155/2015/146365

**Published:** 2015-09-30

**Authors:** Ru Shen, Xiaosheng Wang, Chittibabu Guda

**Affiliations:** ^1^Department of Genetics, Cell Biology and Anatomy, University of Nebraska Medical Center, Omaha, NE 68198, USA; ^2^Bioinformatics and Systems Biology Core, University of Nebraska Medical Center, Omaha, NE 68198, USA; ^3^Department of Biochemistry and Molecular Biology, University of Nebraska Medical Center, Omaha, NE 68198, USA; ^4^Fred and Pamela Buffet Cancer Center, University of Nebraska Medical Center, Omaha, NE 68198, USA

## Abstract

*Background*. The molecular profiles exhibited in different cancer types are very different; hence, discovering distinct functional modules associated with specific cancer types is very important to understand the distinct functions associated with them. Protein-protein interaction networks carry vital information about molecular interactions in cellular systems, and identification of functional modules (subgraphs) in these networks is one of the most important applications of biological network analysis.* Results*. In this study, we developed a new graph theory based method to identify distinct functional modules from nine different cancer protein-protein interaction networks. The method is composed of three major steps: (i) extracting modules from protein-protein interaction networks using network clustering algorithms; (ii) identifying distinct subgraphs from the derived modules; and (iii) identifying distinct subgraph patterns from distinct subgraphs. The subgraph patterns were evaluated using experimentally determined cancer-specific protein-protein interaction data from the Ingenuity knowledgebase, to identify distinct functional modules that are specific to each cancer type.* Conclusion*. We identified cancer-type specific subgraph patterns that may represent the functional modules involved in the molecular pathogenesis of different cancer types. Our method can serve as an effective tool to discover cancer-type specific functional modules from large protein-protein interaction networks.

## 1. Background

PPI networks represent the cross talk among groups of proteins, which have a wide range of biological implications [1, 2]. Computational analysis has become an indispensable tool in understanding the functional significance of PPI networks, given the large volumes of PPI data available from systems biology experiments. Specifically, graph theory based computational methods have been widely used to analyze PPI networks [3, 4]. For example, graph kernels and graph alignments have been used to compare similarities between networks [5]; and graph-clustering and module detection have been used to identify functional modules in PPI networks [6]. For a thorough description of different graph-mining algorithms that have been applied to study biological interaction networks, please refer to a recent review [7].

In a previous study [8], we collected differentially expressed genes (DEGs) between tumor and normal samples from microarray studies of nine different solid tumor types, using the Oncomine database [9]. We constructed nine cancer-type specific PPI networks by mapping DEGs to PPIs of five human protein interactome databases including IntAct [10], MINT [11], HPRD [12], DIP [13], and BIND [14]. We studied the commonality among the nine PPI networks and identified the common modules that frequently occur in these networks. These common modules could be functionally important as they were frequently identified in multiple cancer types. In fact, these modules have been closely associated with cancer-related processes such as transcriptional regulation, cell growth, and cell proliferation [8]. While finding common functional modules (subgraphs) that exist among many cancer types was very useful, it is more valuable to find the modules that are specific to only one cancer type. In contrast to our previous study, this study is focused on discovering distinct cancer-specific functional modules that could offer direct targets for effective drug discovery. Distinct modules are those that exist exclusively in one network and can be discovered by finding distinct patterns in PPI networks. From the graph theory perspective, identification of distinct patterns is differential from identification of common patterns, in that the latter converges as the size of modules increase, while the former diverges.

Existing algorithms, such as RNSC (Restricted Neighbourhood Search Clustering), are effective in extracting modules from networks (more details on the existing algorithms are provided in Supplementary File 1) (see Supplementary File 1 in Supplementary Material available online at http://dx.doi.org/10.1155/2015/146365). RNSC is a local search-based, graph-clustering algorithm that defines a naïve cost function and a scaled cost function, resulting in the lowest clustering cost among comparable methods [15]. Starting from an initial random clustering, RNSC moves vertices among different clusters in order to reduce the cost. RNSC maintains a list of moves referred to as Tabu list, which should be avoided to speed up the process. Once the modules are extracted, it identifies distinct modules that exist only in one network but not in the others. Subgraph query algorithms are used to determine whether a module exists in a given network. Such methods require a subgraph isomorphism test, and as a result querying is computationally expensive. SPath is a subgraph query method [16], which maintains a neighborhood signature (NS) consisting of a group of node sets indexed by shortest path distance, for each vertex. During the subgraph query, NS of the vertices are used to generate the shortest paths of the query graph. A few of the shortest paths are selected to represent the whole query graph. Another approach is graph indexing, which is frequently used as an optimization technique in graph-mining. GraphGrep [17] is a graph indexing algorithm that enumerates all the paths up to a certain length in a network and indexes them as a means to later identify every graph that contains all the paths. Yan et al. proposed a method for quick graph indexing and pattern search known as gIndex [18], which performs graph-based indexing instead of path-based indexing. It uses discriminative fragments to index the networks and is therefore suitable for complex query graphs.

In this study, we developed a new graph theory based method to identify distinct modules between the nine PPI networks, where each network belongs to a distinct cancer. We divided the task into three steps: (1) We used RNSC [15], a local search algorithm that divides networks into nonoverlapping substructures to identify modules in networks. (2) We found distinct subgraphs among the identified modules. And (3) we extracted patterns from the distinct subgraphs and searched for these patterns in other networks. If a pattern does not exist in other networks, we defined it as a distinct module. Using this method, we identified distinct modules or subgraphs that are unique to a given cancer type. We also verified if the unique subgraphs indeed represent PPI networks in specific cancer types using quantitative validation methods. To our knowledge, this work represents the first attempt to identify distinct functional modules in cancer using large-scale PPI networks and graph theory based algorithms.

## 2. Methods

Our method includes three steps: module detection using RNSC, distinct subgraph identification, and distinct pattern identification. We first introduce preliminary concepts and then explain the details of each step in the methodology.

### 2.1. Graph Theory Preliminaries

*Graph*. A graph is a pair *G* = (*V*, *E*), where *V* is the node set and *E*⊆*V* × *V* is the edge set.

*Labeled Graph*. A labeled graph is a triple *G* = (*V*, *E*, *μ*), where *V* is the node set, *E*⊆*V* × *V* is the edge set, and *μ* is the function assigning labels to vertices.

*Graph Isomorphism*. Given two graphs *G* = (*V*, *E*) and *G*′ = (*V*′, *E*′), graph isomorphism is a bijective function *f* : *V* → *V*′ such that ∀*v*_*i*_, *v*_*j*_ ∈ *V*, (*v*_*i*_, *v*_*j*_) ∈ *E*↔(*f*(*v*_*i*_), *f*(*v*_*j*_)) ∈ *E*′.

*Subgraph Isomorphism*. Given two graphs, *G* and *h*, if there exists a subgraph *g* in *G* such that *g* is graph isomorphic to *h*, then *h* is subgraph isomorphic to *G*.

*Graph Patterns*. Given a labeled graph *G* = (*V*, *E*, *μ*), the graph pattern of *G* is an abstraction graph *P* = (*T*, *E*) such that *T* = {*μ*(*v*) : *v* ∈ *V*}. The graph pattern is a special case of the graph isomorphism. When the bijective function in the graph isomorphism is defined to be the assignment of same vertex labels, graphs that belong to the same patterns are isomorphic to each other.

### 2.2. Module Detection Using RNSC

We used RNSC algorithm to generate modules for each of the nine cancer PPI networks. RNSC divides a graph into nonoverlapping connected components, each of which is defined as a module. The results of RNSC clustering depend on the parameter setting. We set up the following parameters for our RNSC runs.

(1) *Tabu list tolerance*: Tabu list stores the vertex moves that should be avoided. Tabu list tolerance is the number of times a vertex must appear in the Tabu list before it becomes forbidden to move the vertex. We chose 1 for this value.
(2) *Tabu length*: the number of items that are stored in a Tabu list (we set it to 50).
(3) *Naive stopping tolerance*: the number of steps the naive scheme will continue without improving the best cost. It determines when to stop running for the naive scheme (we set it to 15).
(4) *Scaled stopping tolerance*: the number of steps the scaled scheme will run without improving the best cost (we set it to 15).
(5) *Diversification frequency*: it represents the shuffling diversification frequency or the destructive diversification frequency, depending on which diversification scheme is used (we set it to 50).
(6) *Shuffling diversification length*: the number of moves for shuffling diversification. If this parameter is set, shuffling diversification will be performed instead of destructive diversification (we set it to 3).

### 2.3. Distinct Subgraph Identification

Distinct modules are not only the unique subgraphs, but also the unique subgraph patterns (a subgraph can have many patterns based on the edge topology) in networks. From the modules generated by RNSC, we searched for those that exist uniquely in each network. We used canonical labels [8] to represent subgraphs in order to quickly identify distinct subgraphs.

### 2.4. Module Labeling

**Figure 1:**
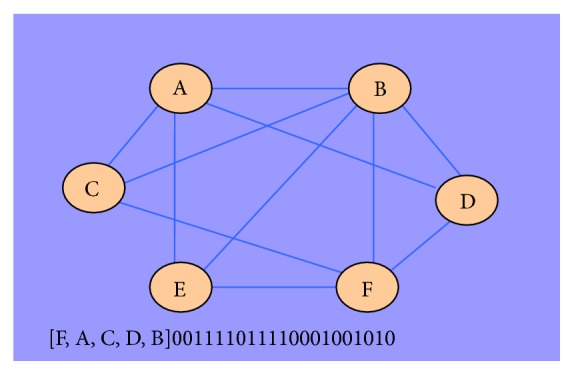
Canonical labeling of a subgraph. The label at the bottom of the figure includes the list of nodes sorted in a given order [in square brackets] followed by the concatenated adjacency matrix.

In McKay's canonical graph labeling algorithm [19], the concept of canonical labeling for graphs was introduced. The basic idea is to represent relational graph data using a sequence of symbols that can uniquely identify a graph. Conversely, a graph must be able to be converted to the same sequence of symbols all the time. Koyuturk et al. proposed to use the concatenation of upper triangle of adjacency matrix as the canonical label of graphs [20]. For a graph without edge weights, its adjacency matrix is a binary matrix in which every row or column corresponds to a node in the graph. The value at the row *i* and column *j* of the matrix is “1” if there is an edge connecting node *i* with node *j*, and “0” otherwise. For an undirected graph, its adjacency matrix is symmetric on the main diagonal. Therefore, we can use the upper right triangle of the adjacency matrix to fully represent a graph. An example of the subgraph labeling is shown in Figure 1.

### 2.5. Distinct Subgraphs

The network modules generated by RNSC may only contain one node. When we identified distinct modules, we set the threshold of minimum number of edges contained in a module as three, considering the smaller the node or edge size the lesser the distinctness. We built a hash table for each network that stores the mapping between the canonical labeling and the actual subgraph. For the modules in each network, we filtered out those that also appear in other networks. We also filtered out the modules that are subgraphs of other modules based on the edge set enclosure.

### 2.6. Distinct Pattern Identification

To label the graph nodes in a PPI network, we used a sequence alignment algorithm to cluster protein sequences into mutually exclusive groups [21]. Proteins present in the same cluster were deemed functionally similar to each other and were assigned the same label. We used stringent criteria of 90% sequence identity over 95% of the length of each sequence and reduced the original set of 18,888 proteins to 14,838 clusters. All proteins in the given cluster contain the same label prefix. For example, cluster *a* containing *n* number of proteins is labeled as *a*_1_, *a*_2_,…, *a*_*n*_. The total number of proteins is the union of all proteins from all cancer networks, so each network contains a subset of these proteins.

**Figure 2:**
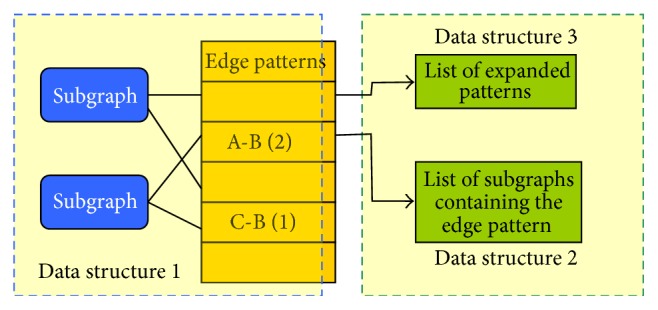
Data structures for distinct pattern identification. Data structure 1 stores the mapping between subgraphs and the edge patterns contained in the subgraphs. Data structure 2 stores the reversed indices from edge patterns to subgraphs containing the patterns. Data structure 3 stores expanded patterns for given patterns.

A graph pattern is the abstraction of graphs created by maintaining the same topology and vertices. In order to facilitate the pattern comparison, we created three data structures as depicted in Figure 2. The first data structure stores list of edge patterns for each subgraph. Edge patterns are the edges in the subgraphs, with vertices replaced by labels. Since our PPI networks are undirected graphs, the order of vertices in edge labels was not considered when assigning patterns. We added a number to the end of the edge pattern to indicate how many times that edge pattern occurred in the graph. For example, edges a1-b1 and b2-a5 belong to the same pattern, A-B [8]. If a graph has three edges of A-B pattern, it will point to A-B (3) edge pattern. The second data structure maintains a list of subgraphs that contain the edge patterns. Similar to the graph indexing technique used in GraphGrep [17], the second data structure is a reverse index from the edge pattern to the subgraphs. It can speed up the searching of subgraphs to a greater extent. The third data structure stores the expanded patterns for each edge pattern. Expanded patterns are the edge patterns with the same gene combination but higher count; that is, for edge pattern A-B (2), the expanded patterns are all A-B (*k*) patterns, where *k* > 2.

With the three data structures, we can largely reduce the number of potential matching subgraphs for a given subgraph. Given a subgraph, we performed some preliminary filtering based on its number of nodes and number of edges. Subgraphs from other networks were filtered out if their number of nodes or edges is smaller than that of the given query subgraph. We got the query subgraph's edge patterns from the first data structure and then expanded the edge patterns by supplementing their expanded patterns. For example, if A-B (2) is in the edge pattern, then we will include all A-B (*k*) patterns, where *k* > 2, to the pattern list. The purpose of the pattern expansion is to find subgraphs that contain the query graph pattern as a subgraph. The expanded edge patterns were used to search matching subgraphs. For each edge pattern of the query subgraph, we got the list of subgraphs containing the pattern based on the second data structure and then intersected the subgraphs to obtain the list of subgraphs that may potentially match the pattern of the query graph. If the resulting list is empty, the query subgraph has a distinct pattern and therefore is a distinct module. If the resulting list is not empty, further verification is required to examine whether the query subgraph really matches the discovered subgraphs. The pseudocode for the algorithms is given in Algorithms 1 and 2.

We verified whether or not the query subgraph and the matching subgraphs contain the same edge patterns based on node information. From the query subgraph, we selected the node with the highest degree and looked for its counterpart in the matching subgraphs. If the counterpart does not exist in one matching subgraph, we filtered out the subgraph. Otherwise, we extended the search to look for the next node that is connected to the previous node and had the highest degree. The process was halted whenever a node from the query subgraph could not be matched to any node in the other subgraph. If all nodes in the query graph were mapped to their counterparts in the other subgraph, then we found a truly matching subgraph of the query subgraph. For a query subgraph, if all of its matching subgraphs were filtered out, then this query graph was included into the distinct module set. Since the subgraphs with less than three edges do not contain enough interaction information, we used only those subgraphs with three or more edges for further analysis.

The running time and resource requirements for Algorithm 2 are very high, as it tries to match subgraphs node by node. This is similar to a depth-first search, but without the backtrack process. However, Algorithm 2 runs only on a limited set of subgraphs since Algorithm 1 has effectively filtered out all the nonmatching subgraphs, leaving only a few potential candidates. This helps reduce the overall running time of the method.

### 2.7. Calculation of GO Semantic Similarity

The semantic similarity of GO terms between two interacting proteins was calculated for all possible pairs of proteins in the human PPI network. The GO terms associated with each protein were obtained from the GO database. The GO annotation (GOA) for a protein can be based on three concepts: biological process (P), molecular function (F), and cellular component (C). The best semantic similarity measure between the GO terms of the two proteins, under each GO concept, was determined for all pairs of proteins using the method proposed by Brown and Jurisica [22].

The probability of minimum subsumer, *P*_ms_, was determined separately for biological process (P) and molecular function (F) and cellular component (C) using the following derivation: (i) Let *g*_*i*_ and *g*_*j*_ represent the set of GO terms from proteins *i* and *j*, respectively; (ii) let *S*(*g*_*i*_, *g*_*j*_) represent the set of shared parental GO terms of *g*_*i*_ and *g*_*j*_; (iii) let *G*_*c*_ represent GO concept P, F, or C; and (iv) let *g*_*p*_ be a shared parental GO term. Then, *P*_ms_ is calculated as the probability of minimum subsumer (the least frequent of all the parental GO terms in the set), over each concept. Consider

(1) $$\begin{array}{c} P_{\text{ms}}\left(\;g_{i},g_{j}\;\right)=\underset{S\left(g_{i},g_{j}\right)\;|\;G_{c}}{\min}\left\{\;p\left(\;g_{p}\;\right)\;\right\}. \end{array}$$

A similarity measure based on this probability is then calculated as the negative log probability of minimum subsumer, using the following equation:

(2) $$\begin{array}{c} \text{Sim}\left(\;g_{i},g_{j}\;\right)=-\ln\left(\;P_{\text{ms}}\left(\;g_{i},g_{j}\;\right)\;\right). \end{array}$$

The similarity score between a pair of GO terms is higher if they share a common parent containing more specific GO term (less frequent), and vice versa. The total similarity score is the sum of the best similarity scores from each concept.

### 2.8. Validation of the Cancer-Type Specific Distinct Subgraph Patterns

We used the IPA (Ingenuity Systems, http://www.ingenuity.com/) PPI data to validate the cancer-type specificity of the distinct subgraph patterns that we generated in this study. IPA is a system that yields a set of networks relevant to a list of genes based on the curated records contained in the Ingenuity Pathways Knowledge Base (IPKB), which were constructed by collecting experimental evidence published in literature. When a list of genes is fed into IPA, its core analysis tool maps the gene list to the IPKB and generates molecular interaction networks that are most likely relevant to the input gene list. We input all the nodes in the distinct subgraphs relevant to each cancer PPI network into IPA and generated the human cancer-type specific PPI networks by selecting appropriate parameters. These parameters include “Human” in the “Species” options and specific-type of cancer cell lines in the “Tissues and Cell Lines” options. We generated six PPI networks related to breast, cervical, colorectal, melanoma, pancreatic, and prostate cancers. We could not generate PPI networks related to bladder, esophagus, and gastric cancers because IPA does not have these three cancer types listed in the “Tissue and Cell Lines” options. Finally, we mapped our distinct subgraphs to the IPA generated networks to validate if corresponding subgraphs are indeed cancer-type specific.

## 3. Results and Discussion

### 3.1. Cancer Protein Interaction Networks

Cancer PPI networks were constructed from a comprehensive, nonredundant dataset of experimentally derived PPIs that were collected from five major databases including IntAct [10], MINT [11], HPRD [12], DIP [13], and BIND [14]. Since PPI data that are specific to a cancer type do not exist in the public domain, we used all the available PPI datasets for humans from five major databases as the basis for our studies. In our final human PPI network, there are 19,710 unique proteins representing 95,931 unique interactions. Note that this unique set of proteins exhibit some level of redundancy because splice variants with minimal sequence differences are included as unique proteins due to the fact that PPIs are isoform-specific.

We collected differentially expressed genes (DEGs) between tumor and normal samples from microarray studies of nine different solid tumor types using the Oncomine database [23]. Oncomine is a cancer microarray database that provides access to DEGs on most major types of cancer. For each type of cancer, DEG lists are available from multiple experiments, where the *q*-values (a variant of *P* value) for a gene vary from experiment to experiment. Therefore, we chose only DEGs whose average *q*-values are equal to or smaller than 0.05. The gene lists were then mapped to protein lists using our in-house mapping tools. The number of proteins is roughly two times the number of genes due to the multiple mappings between genes and proteins. These proteins were further mapped to the proteins in the human PPI network to create nine cancer-specific PPI networks.  Table 1 summarizes the number of genes and proteins and the corresponding network size associated with each cancer type.

### 3.2. Identification of Distinct Modules, Subgraphs, and Patterns from Cancer PPI Networks

Distinct subgraphs and subgraph patterns are those that exist in only one cancer PPI network but not in the others. A distinct pattern may contain multiple distinct subgraphs; that is, there is one-to-many relationship between a distinct pattern and distinct subgraphs. To find the distinct patterns, we first identified all distinct subgraphs and then extracted patterns from them. In the worst case, the number of distinct subgraphs is *O*(*d* ^*k*^), where *k* is proportional to the number of edges (*d*) in networks. Because the computational complexity in this case is intractable, an alternative way to make this tractable is to first identify the modules and then find distinct modules. We obtained a large number of modules for each of the nine cancer PPI networks using RNSC.  Table 1 shows cancer PPI network statistics, the number of modules generated for each cancer PPI network, and also the number of corresponding distinct subgraphs and patterns that would be generated in the subsequent steps. Figure S3 in Supplementary File 1 shows examples of multiple distinct subgraphs that map to a distinct pattern.

From the network modules generated by RNSC, we identified hundreds of distinct subgraphs for each of the nine cancer PPI networks (Table 1) by filtering out those that also appear in other networks, including those that are subgraphs of other modules based on the edge set enclosure. We identified distinct subgraph patterns by comparing and filtering out graph patterns that have the same topology and vertices (or those with the same cluster label). Some of them, however, have instances of the same pattern appearing in multiple networks. For distinct pattern identification, we selected those patterns that are only occurring in one network as modules. We did not select those that are occurring in other networks, either as modules or as subgraphs of modules. The total numbers of modules, distinct subgraphs, and distinct subgraph patterns for each cancer PPI network are shown in Table 1. The numbers of distinct subgraphs and distinct patterns generated in each cancer type are proportional to the number of modules obtained in corresponding cancer networks, suggesting that each cancer type has its own set of functional processes that are carried out through different number, type, and topology of interacting proteins.

**Figure 3:**
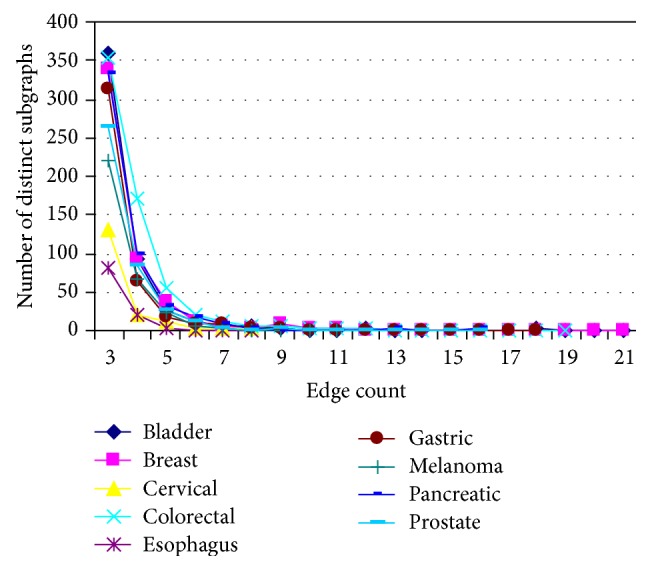
Size distribution of distinct subgraphs in the nine cancer PPI networks. The *x*-axis represents the size of subgraphs (number of edges), and the *y*-axis represents the number of subgraphs at each size.

**Figure 4:**
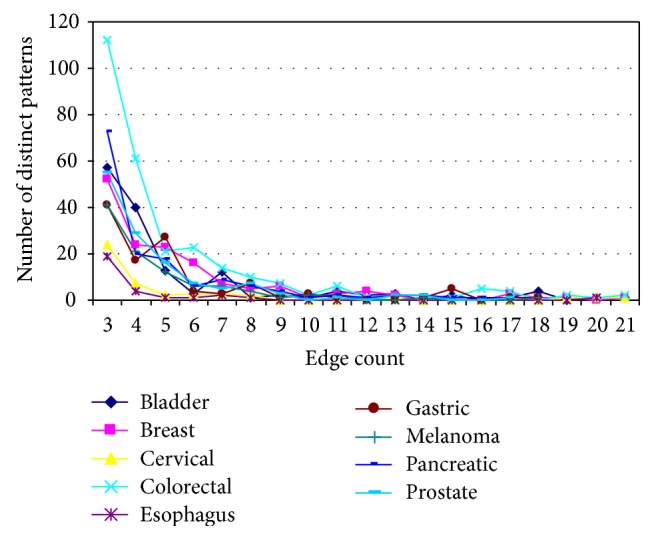
Size distribution of distinct patterns in the nine cancer PPI networks. The *x*-axis represents the size of subgraph patterns (number of edges), and the *y*-axis represents the number of subgraph patterns at each size.

Figures 3 and 4 show the size distribution of distinct subgraphs and distinct patterns in the nine cancer PPI networks, as a function of their edge count. The number of distinct subgraphs declines quickly from 3- to 5-edge subgraphs and almost flattens out beyond 5 edges for all the nine cancer types (Figure 3). The number of distinct patterns (Figure 4) follows a similar trend, except that they show some variation across different cancers until the patterns reach 9 edges. Obviously, the most frequently occurring subgraphs and patterns are 3-edge subgraphs across all the cancer networks. These observations indicate that most of the distinct subgraphs and distinct patterns in different cancers are formed by a smaller number of interacting partners (with only 3–5 edges) that can be easily associated and dissociated in the cellular environment.

### 3.3. Biological Relevance of Distinct Patterns

**Figure 5:**
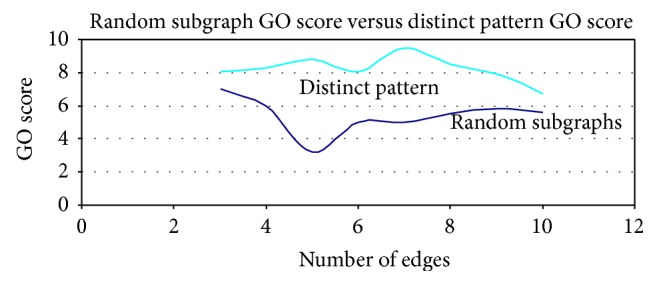
Comparison of GO semantic similarity score. The GO semantic similarity score of distinct patterns is consistently higher than random subgraphs.

To determine if the identified subgraph patterns are biologically meaningful or not, we compared the semantic similarity of Gene Ontology (GO) terms corresponding to the interactions (edges) in the subgraph patterns against those from the randomly generated subgraph patterns in the same *n*-edge group, where *n* varies from 2 to 12. Semantic similarity [22] provides a quantitative measure (with a score range of 0–10) of how similar a pair of proteins is, based on the GO annotations. Because the interacting proteins are more likely associated with similar cellular processes and/or involved in similar function, this similarity measure is higher for functionally related proteins, and vice versa. This concept has been very effective in interpreting the functional similarities of genes/proteins based on gene annotation information from heterogeneous data sources [8, 24]. As shown in Figure 5, the GO semantic similarity score of distinct patterns is consistently higher than the randomly generated subgraphs at all *n*-edge groups, suggesting that the identified subgraph patterns are biologically relevant.

### 3.4. Validation of Cancer-Specific Distinct Subgraph Patterns

**Figure 6:**
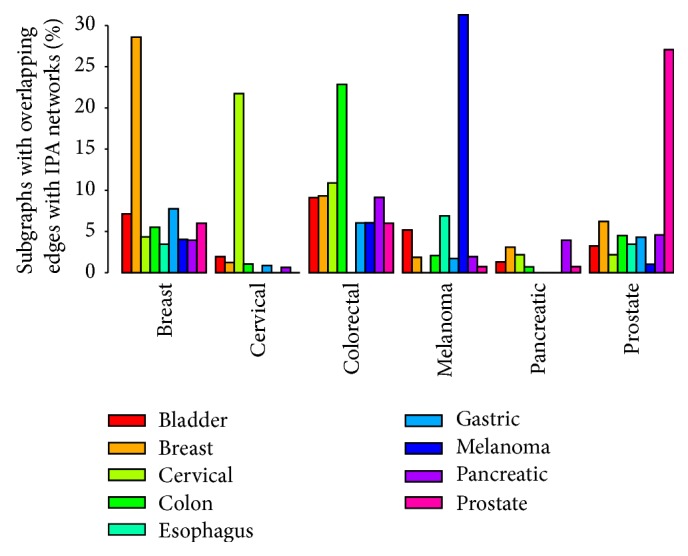
Distribution of distinct subgraphs in PPI networks across the IPA cancer-specific networks.

In this experiment, we validated the distinct subgraph patterns identified in our study against experimentally known caner-specific PPI networks obtained from the Ingenuity Pathway Analysis (IPA) Knowledge Base. Cancer-specific network information was not available for bladder, esophagus, and gastric cancers from IPA; therefore we used only six cancer networks for the validation study.  Figure 6 shows the distribution of distinct subgraphs across different cancer-specific networks based on the percentage of the distinct subgraphs that have at least one overlapping edge with each of the six IPA cancer networks. Because the available PPI data is incomplete, we counted those subgraph patterns that have at least one overlapping edge in a cancer-specific network. It is expected that the group of distinct subgraphs from a given cancer type will have more overlapping edges in its corresponding IPA cancer network but less overlap in the dissimilar networks. As seen in Figure 6, the distinct subgraph patterns from all the PPI cancer networks are highly enriched in the corresponding IPA cancer networks compared to other cancers. It is also evident that unlike all the other cancers, the distinct subgraphs in pancreatic cancer are generally not highly enriched despite having one of the highest numbers of distinct subgraphs and subgraph patterns (Table 1) in this cancer type. The reason for this could be due to the IPA pancreatic cancer network having much smaller scale compared to almost all the other caner networks (there are 921, 71, 1689, 346, 52, and 549 edges contained in the IPA breast, cervical, colorectal, melanoma, pancreatic, and prostate cancer networks, resp.).

As the sizes of *n*-edge subgraph patterns vary, we defined the edge overlapping rate of a subgraph with an IPA network as the ratio of the number of overlapping edges to the total number of edges in a subgraph. We calculated the edge overlapping rate for all the distinct subgraphs in the six IPA networks (as shown in Supplementary File 2). For each cancer, we carried out a one-sided *t*-test by comparing the overlapping rates of subgraphs from the same cancer versus those from all the other cancers (with the hypothesis that the distinct subgraphs from a given cancer will have higher edge overlapping rate in the IPA network of the same cancer type).  Table 2 lists all the *t*-test *P* values, showing that overlapping rates of subgraphs from dissimilar cancers are significantly lower than those from the same cancer (*P* value < 0.05) with the exception of two cases in pancreatic cancer. Again, the dismal performance of subgraph patterns in pancreatic cancer may be attributed to the lack of sufficient cancer-specific data for this cancer in the IPA network. Since the IPA networks were constructed based on experimental evidence, the significantly lower overlapping rates of edges from distinct subgraphs of different cancer types indicate that the distinct subgraph patterns we identified are cancer-type specific.

Figure S4 in Supplementary File 1 shows one example of a caner-type specific PPI module corresponding to each of the breast, cervical, colorectal, melanoma, pancreatic, and prostate cancers based on the IPA data. These patterns are worthy of experimental verification in corresponding cancers since experimental evidence that supports the cancer-specificity of these patterns is insufficient.

## 4. Conclusions

In this study, we developed the methodology to extract the distinct functional modules from nine cancer-specific PPI networks. In order to identify distinct modules we employed a 3-step strategy. The first step is to search for modules in the networks. We used RNSC, a local search algorithm, to divide each network into nonoverlapping partitions based on the network's connectivity. In the second step, distinct subgraphs that uniquely exist in single networks were identified from the modules discovered in the first step. In the third step, we filtered the distinct subgraphs to keep only those that have unique patterns across the networks. We implemented canonical labeling to expedite the identification of unique subgraphs in the second step and graph indexing for fast retrieving of subgraphs based on edge patterns in the third step.

The subgraph patterns identified in this study are more biologically significant (as measured by the GO semantic similarity) when compared to the subgraph patterns that are randomly generated from the cancer-specific networks (Figure 5). Validation of distinct subgraph patterns against cancer-specific IPA networks (experiment-based evidence) showed high correspondence between identical cancer types, indicating that the distinct subgraph patterns we identified are likely to be cancer-type specific. As new PPI data emerge, we hope to use our method to identify cancer-type specific functional modules that may contribute to specific molecular pathogenesis of different cancer types. In addition, the methodology developed in this study can also be applied to study the PPI networks from other diseases.

## 5. Glossary

*Distinct Modules*. When performing comparative analysis on multiple protein-protein interaction networks, we define distinct modules as the functional modules that exist exclusively in a subset of protein-protein interaction networks.

*Distinct Subgraphs*. From the given protein-protein interaction networks, we identify subgraphs that are significant according to our clustering algorithm. The subgraphs that exist exclusively in a subset of networks are distinct subgraphs.

*Distinct Patterns*. Patterns are abstraction of graphs. In the context of this research, subgraph patterns have the same topology as the subgraphs but with the nodes replaced by cluster label of the original nodes. In this way, different subgraphs may belong to the same pattern if they share the same topology and similar nodes. Distinct patterns refer to a stricter concept than distinct subgraphs, because distinct subgraphs may not belong to distinct patterns if they share topology with other subgraphs.

## Supplementary Material

Supplementary File 1 contains more details on the existing graph clustering algorithms and supplementary figures.
Supplementary File 2 contains the edge-overlapping rates for all the distinct subgraphs in the six IPA networks.

## Figures and Tables

**Algorithm 1:**
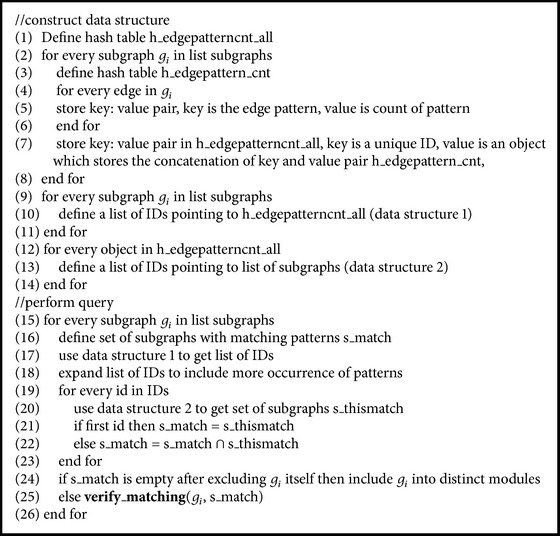
Distinct pattern detection (list of subgraphs).

**Algorithm 2:**
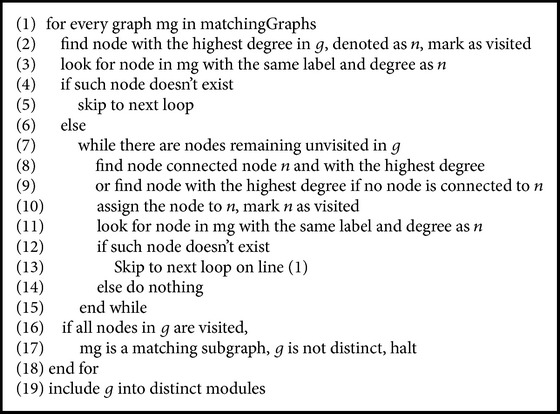
Verify_matching (graph *g*, list of matchingGraphs).

**Table 1:** Number of distinct modules, distinct subgraphs, and distinct patterns generated for each cancer PPI network.

Cancer PPI Network	Protein count	Edge count	Node count	Number of modules	Number of distinct subgraphs	Number of distinct patterns
Bladder cancer	29286	47909	10726	5129	510	154
Breast cancer	26498	33558	8611	6565	508	161
Cervical cancer	22447	19332	6288	1144	167	46
Colorectal cancer	40905	58212	13273	6357	638	289
Esophagus cancer	13380	13405	4218	767	103	29
Gastric cancer	28224	41289	9707	4038	425	116
Melanoma cancer	22421	30843	7677	2204	322	99
Pancreatic cancer	37160	52125	12199	5581	500	153
Prostate cancer	27598	41658	9621	3070	396	133

**Table 2:** Comparison of edge overlapping rate between two groups of distinct subgraphs.

Nine PPI networks	Six IPA networks					
	Breast	Cervical	Colorectal	Melanoma	Pancreatic	Prostate
Bladder	1.13∗10^−5^	0.0026	1.0∗10^−4^	2.11∗10^−6^	0.048	8.47∗10^−8^
Breast	NA	0.0015	9.7∗10^−4^	1.93∗10^−7^	0.15	7.93∗10^−7^
Cervical	1.71∗10^−7^	NA	0.0039	9.2∗10^−8^	0.173	2.84∗10^−8^
Colorectal	8.6∗10^−4^	0.0014	NA	2.59∗10^−7^	0.033	3.07∗10^−7^
Esophagus	1.72∗10^−5^	0.0016	2.38∗10^−11^	1.07∗10^−5^	0.024	2.45∗10^−5^
Gastric	6.0∗10^−4^	0.0014	1.18∗10^−6^	1.96∗10^−7^	0.024	8.7∗10^−6^
Melanoma	8∗10^−4^	0.0019	1.08∗10^−6^	NA	0.024	6.7∗10^−6^
Pancreatic	2.7∗10^−7^	0.0014	3.65∗10^−5^	7.5∗10^−6^	NA	2.65∗10^−7^
Prostate	1.01∗10^−5^	0.0015	5.08∗10^−8^	7.1∗10^−4^	0.046	NA

Note: for each of the six IPA networks (column), the *t*-test *P* values are shown by comparisons of the edge overlapping rate of distinct subgraphs and the IPA network between the group of distinct subgraphs with the same cancer type as the IPA network and each of the other groups of distinct subgraphs with different cancer types from the IPA network.
