# Supplementary material for: Discovering Distinct Functional Modules of Specific Cancer Types Using Protein-Protein Interaction Networks
Source: Biomed Res Int. 2015 Sep 30;2015:146365. doi: 10.1155/2015/146365 (PMC4606133; doi:10.1155/2015/146365)
Supplement: Supplementary file 1 — contains more details on the existing graph clustering algorithms and supplementary figures. Supplementary File 2 contains the edge-overlapping rates for all the distinct subgraphs in the six IPA networks. [file 146365.f1.zip › SUPPLEMENTARY DATA.pdf]

SUPPLEMENTARY DATA

Supplementary data files can be accessed from the following URLs

http://genome.unmc.edu/ru/SupplementaryFile1.docx

http://genome.unmc.edu/ru/SupplementaryFile2.xlsx
