# Supplementary material for: Discovering Distinct Functional Modules of Specific Cancer Types Using Protein-Protein Interaction Networks
Source: Biomed Res Int. 2015 Sep 30;2015:146365. doi: 10.1155/2015/146365 (PMC4606133; doi:10.1155/2015/146365)
Supplement: Supplementary file 1 — contains more details on the existing graph clustering algorithms and supplementary figures. Supplementary File 2 contains the edge-overlapping rates for all the distinct subgraphs in the six IPA networks. [file 146365.f1.zip › Supplementary File 1 doc.doc]

**Shen R, Wang X and Guda C**

**Supplementary Data**

## More details about related work

### 1. RNSC

RNSC is a local search based graph-clustering algorithm. It uses cost functions to evaluate the result of clustering. An optimal clustering is the one associated with the lowest cost. RNSC proceeds in two steps. In the first step, a naive cost scheme is used to determine the clustering. This step serves the function of preprocessing the graph. In the next step, a scaled cost scheme is used. Several different types of moves are applied on the vertices in order to minimize the clustering cost. Generally more than one experiment will be run. The final output is the one with lowest scaled cost.

The naive cost function is derived from Stijn van Dongen's coverage measure. For a clustering C on a graph G = (V, E) in which |V|=n, the coverage measure is expressed as

where $\#_{out}^{1}(G,C,v)$is the number of external edges incident to v and $\#_{in}^{0}(G,C,v)$is the number of vertices in C_v_ that are not adjacent to v. In a good clustering, both values should be small for all vertices. The cost of clustering on a graph G is expressed as

This cost function is lacking precision because it does not consider the size and density of the original graph. The cost will be always low for sparse graphs regardless of the clustering effectiveness. The scaled cost function normalizes the above cost by size of graphs. The scaled cost function is defined as

N (v) is the open neighborhood of v. C_v_ is the size of the cluster containing v. The factor (n-1)/3 is used to preserve the bound of n-1 in the change of cost of a single move that is present in the naive cost function.

Starting from an initial random clustering, RNSC moves vertex from one cluster to another based on the result of cost functions. If a move decreases the cost, RNSC keeps the new clustering; else it keeps the old clustering. RNSC uses several different types of moves, including global move, diversification move, intensification move and forbidden moves. Global moves are the type of moves that aims at reducing the cost of clustering. Usually the move that results in optimal cost of clustering is picked. For the naive scheme, it is from a set of equivalent cost moves, which incur the same change in cost. For the scaled scheme, the sets include moves whose change in cost rounds up to the same integer. Figure S1 illustrates a global move that aims at reducing the cost of clustering under the naïve scheme.

Figure S1: RNSC global moves

Diversification moves are random moves that purposely shuffle the vertices to avoid circulation around a high local minimum. One diversification scheme is to shuffle vertices randomly for fixed number of steps. The problem with diversification scheme is that the structure of clusters changes very little and vertices tend to move back to their original positions. The other diversification scheme demolishes some of the clusters and moves their vertices randomly to other clusters. The second scheme appears to be more effective than the first one. Intensification moves are like global moves that aim at reducing the cost of clustering. But intensification moves are chosen from a restricted set of moves. RNSC has the moves sorted by their resultant change in cost. So it becomes unnecessary to search through the whole search space to look for a good move. A majority of computational work done by the RNSC algorithm involves not searching, but updating the search space. The last but not least move type is the forbidden move, also called Tabu move. One reason for maintaining a list of Tabu moves is to speed up the process by avoiding unnecessary moves or moves that have happened already. Another reason is to avoid local minimum.

### 2. Subgraph query

The definition of subgraph query problem is: given a network G and a query graph Q, find all matching of Q in G. The subgraph query problem is hard because it requires subgraph isomorphism checking of Q against G, which has been proven to be NP-complete. Here we review several subgraph query algorithms that use different optimization strategies to speed up the process and provide a close-to-optimal solution.

SPath is a subgraph query method proposed by Zhao and Han . SPath maintains for each vertex of the network a neighborhood signature (NS). Given a vertex v in a graph and a nonnegative number k, the NS of v is a group of node sets indexed by shortest path distance from 0 to k. In Figure S2, when k=0, the NS for node 1 written as NS(1) = {{A:{1}}}. When k=1, NS(1) = {{A:{1}},{B:{2}, C:{3}}}. When k=2, NS(1) = {{A:{1}},{B:{2}, C:{3}},{A:{4,6}, B:{5}}}.

Figure S2: Labeling the neighborhood signature

Shortest path information of each vertex is well preserved in neighborhood signature. During query processing, the neighborhood signature is used to reduce search space and find matching of query graph in the target network. The first step of query processing is decomposition. This is the process of identifying NS for each vertex in the query graph. At the same time a list of shortest paths in the query graph are generated. The second step is path selection. In this step, the original query graph is reconstructed by selecting a few shortest paths that cover all edges in the graph. A greedy approximation algorithm is used to pick the smallest set of shortest paths that cover the query graph. The selected shortest paths are used to represent the query graph in the next step of query processing. In the third step, path instantiation is performed, i.e. paths from query graph are tried to match edges in the target network. When selected paths are instantiated and joined with no join-predicates violation, one matching of Q against G is found. The algorithm will not stop until all instances of Q are detected from G.

GraphQL is a graph query language and implementation that support query of graph patterns in large networks . In the GraphQL data model, graphs are the basic unit of information. Node, edge, or graph can have arbitrary attributes. Graph patterns are graphs with predicate specified on the attributes. GraphQL defines graph algebra for the query language along the lines of the relational algebra. A list of operators such as “selection”, “cartesian product” and “composition” are defined. Using the data model, predicates and graph algebra defined in GraphQL language, we can express queries on graph patterns. The general implementation of GraphQL is a depth-first matching. The algorithm consists of two phases. The first phase retrieves all matching nodes in the target network for each node in the query graph, according to the predicates. The search space is the product of all matching of nodes in the graph pattern. The second phase searches in a depth-first manner for matching between the graph pattern and the graph. The worst-case time complexity of the algorithm is exponential to the size of the query graph. Three optimization techniques are used to reduce the running time. The first technique is to index network nodes by its predicate. This avoids a sequential scan of all nodes in a large graph. Matching nodes can be further filtered by neighborhood topology. The second technique reduces the search space by checking for each node whether the adjacent subtree in query graph is sub-isomorphic to that in the target network. An existing algorithm is used in this step. The third technique is to find a good search order according to the cardinality of nodes in the search tree.

Yan et al. proposed gIndex for quick graph indexing and pattern search in networks . Graph indexing is an optimization technique frequently used in graph mining algorithms . GraphGrep , for example, enumerates all the paths in a database up to maxL length and indexes them, where a path is a sequence of vertices. GraphGrep uses the index to identify every graph that contains all the paths (up to maxL length) in query graph. The path-based indexing approach is not suitable for complex graph queries because a path is too simple to preserve the structural information and the number of paths in a network is overwhelming. The gIndex solves the problems by performing graph-based indexing instead of path-based indexing. It uses discriminative fragments to index the networks. Discriminative fragments are the frequent and non-redundant fragments in the network. They expose the intrinsic characteristic of network structures. The gIndex algorithm is divided into four steps: discriminative fragment selection, index construction, searching, and verification. To find discriminative fragments, gIndex first generates all frequent fragments with the size-increasing support constraint, and then distills the fragments to eliminate the redundant ones. In the index construction step, gIndex constructs a prefix tree in which all leaf nodes are discriminative fragments, and records the identities of the graphs containing the discriminative fragments. The searching step takes the intersection of the graphs associated with all discriminative fragments of query graphs. These intersecting graphs are candidates for next step of verification. In the verification step, a subgraph isomorphism test is performed on each graph to check whether the graph really contains the query graph or not.

**Figure S3. Multiple subgraphs of the MYC pattern vary by nodes of the same cluster at an equivalent position.** The 4 subgraphs shown have similar nodes (TUBA4A, TUBA8, TUBA1B and TUBA1A) at corresponding positions. Therefore they belong to the same pattern.

**Figure S4. Examples of distinct modules in different cancer types.**
